# Supplementary material for: The Impact of SIV-Induced Immunodeficiency on SARS-CoV-2 Disease, Viral Dynamics, and Antiviral Immune Response in a Nonhuman Primate Model of Coinfection
Source: Viruses. 2024 Jul 21;16(7):1173. doi: 10.3390/v16071173 (PMC11281476; doi:10.3390/v16071173)
Supplement: Supplementary file 1 [file viruses-16-01173-s001.zip › viruses-3091106-supplementary.pdf]

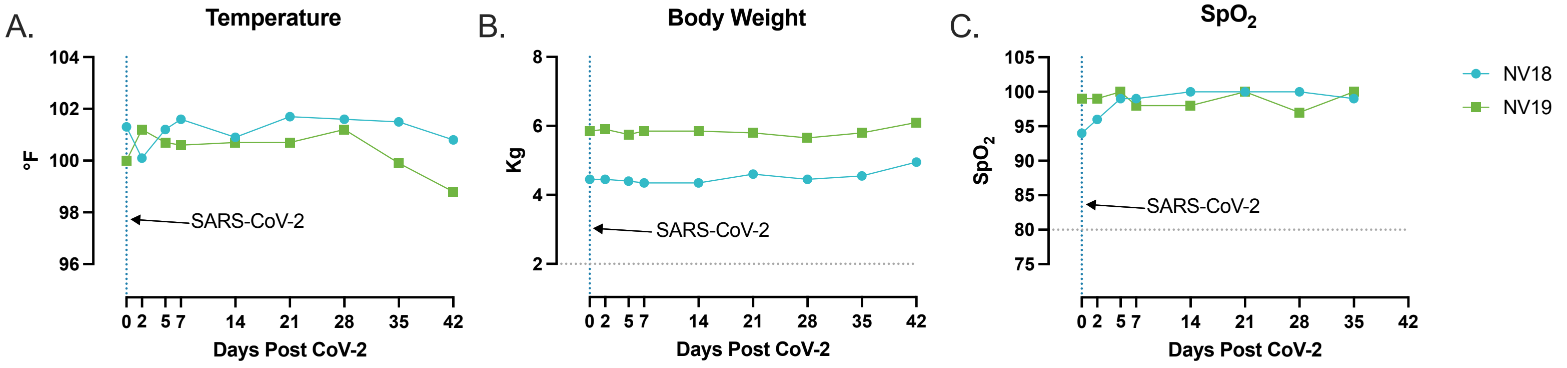

Supplemental Figure S1. Temperature (**A**), weight (**B**), and saturation of peripheral oxygen (SpO2) (**C**) levels were measured prior to and for 6 weeks following SARS-CoV-2 inoculation of SIV+ pigtail macaques. Day 0 indicates time of SARS-CoV-2 infection, 48 weeks post SIVmac239 exposure.

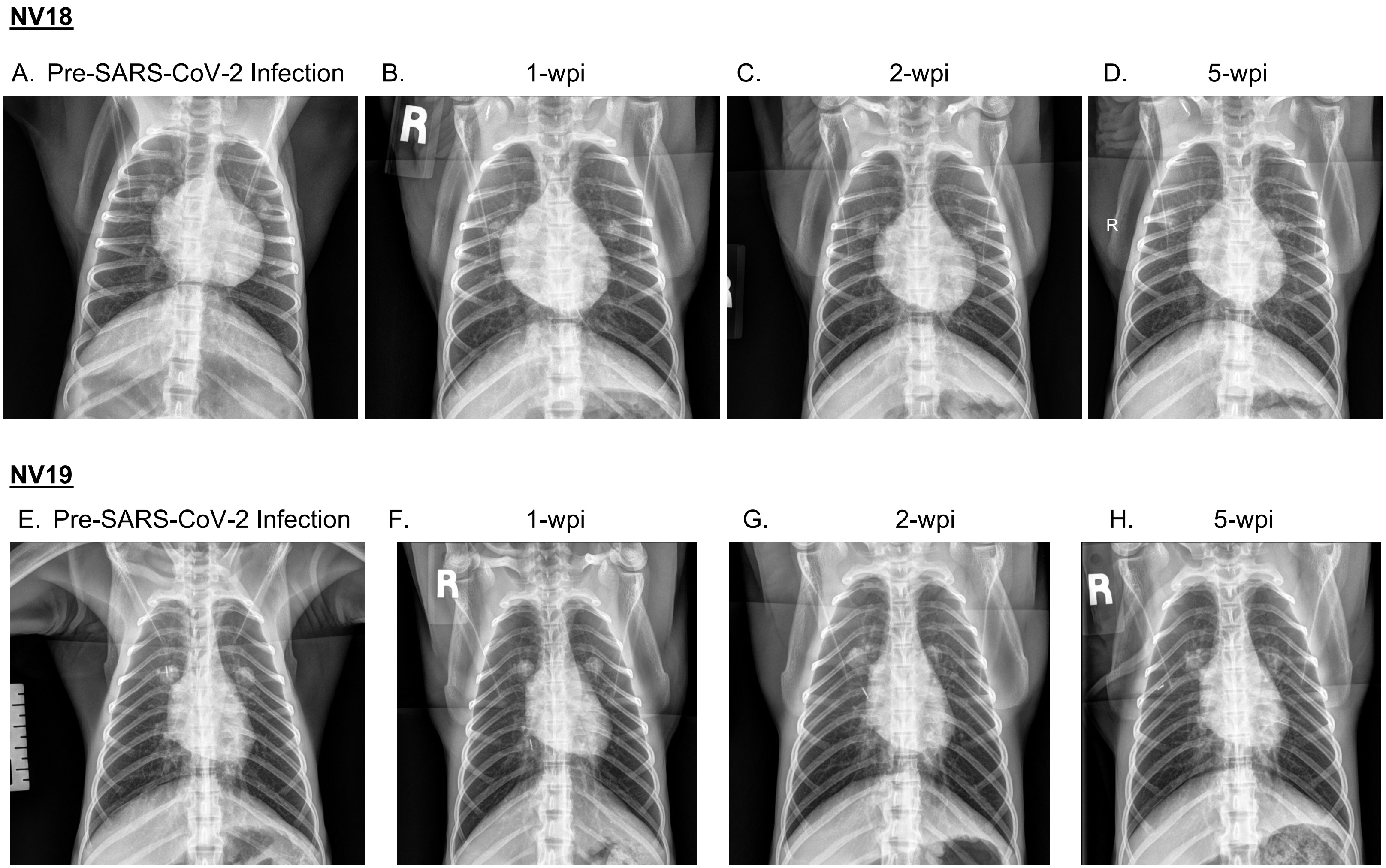

Supplemental Figure 2. Radiographs of SIV-infected pigtail macaques (PTM) challenged with SARS-CoV-2. Radiographs were obtained prior to SARS-CoV-2 infection and at weeks 1-, 2-, and 5-weeks post infection (wpi). Baseline was established at 2 weeks prior to SARS-CoV-2 inoculation.

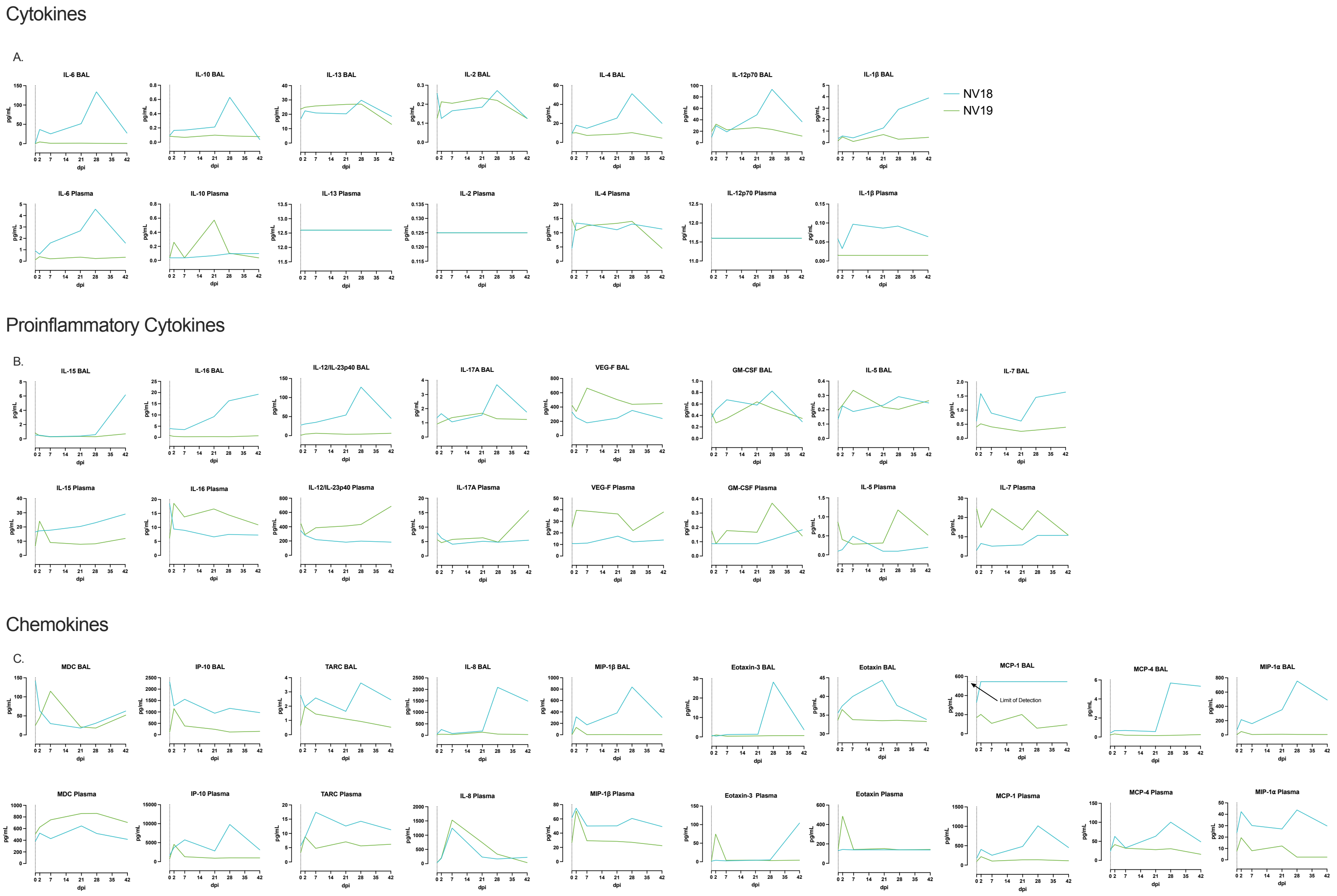

Supplemental Figure 3. Meso Scale analysis of cytokine and chemokine fluctuations in blood and BAL in PTM coinfected with SIV and SARS-CoV-2. **A-C.** Line graphs illustrating cytokine, proinflammatory cytokine, and chemokine dynamics in BAL supernatant and plasma before and 2-, 7-, 21-, 28-, and 42-days post SARS-CoV-2 infection.

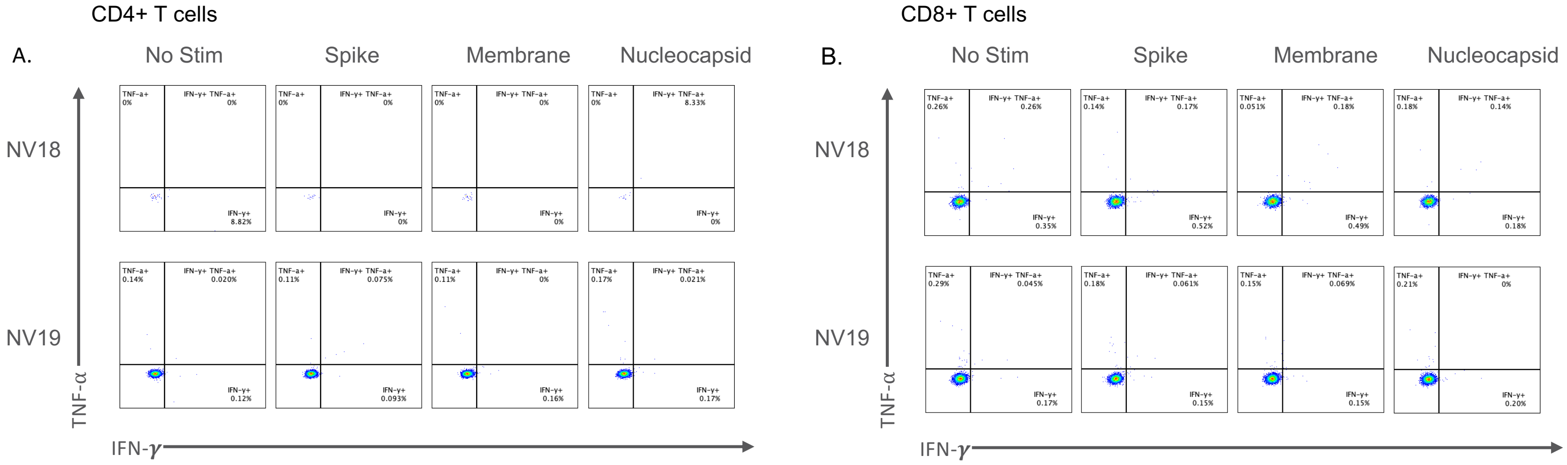

Supplementary Figure 4. Peripheral SARS-CoV-2 specific T cell responses were undetectable 21-days-post-infection. Two female pigtail macaques (PTM, NV18 & NV19) co-infected with SIVmac239 and SARS-CoV-2 shown. **A&B**. Flow cytometry dot plots demonstrating the IFN-y and TNF-a response of CD4+ (**A**) and CD8+ (**B**) T cells to overnight SARS-CoV-2 peptide (spike, membrane, and nucleocapsid) stimulation. No Stim = cells incubated overnight without peptide stimulation. Note: NV18 exhibited virtually no detectable CD4+ T cells in blood at the time of sampling, thus the empty flow plot for CD4+ T cell cytokine response to stimulation.

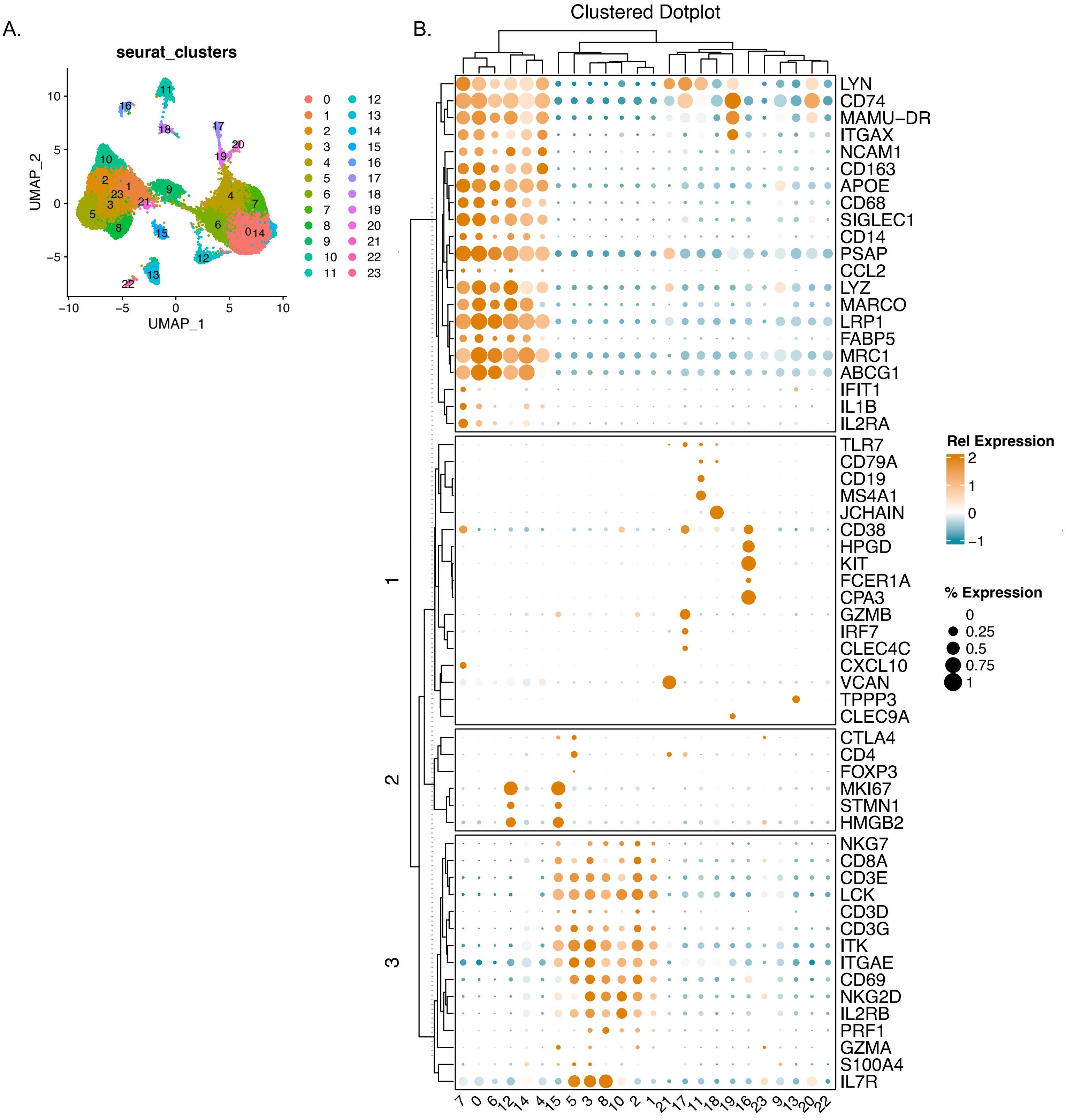

Supplementary Figure 5. Defining Seurat Clusters. A. UMAP displaying Seurat-derived clusters obtained from FindAllMarkers function in Seurat. **B.** Dot plot depicting markers used to identify cell types. Hierarchical clustering method (hclust) was used to cluster columns and rows. The color of the dots indicates the relative gene (Rel. Expression). Dot size represents the percentage of cells expressing the gene (% Expression).

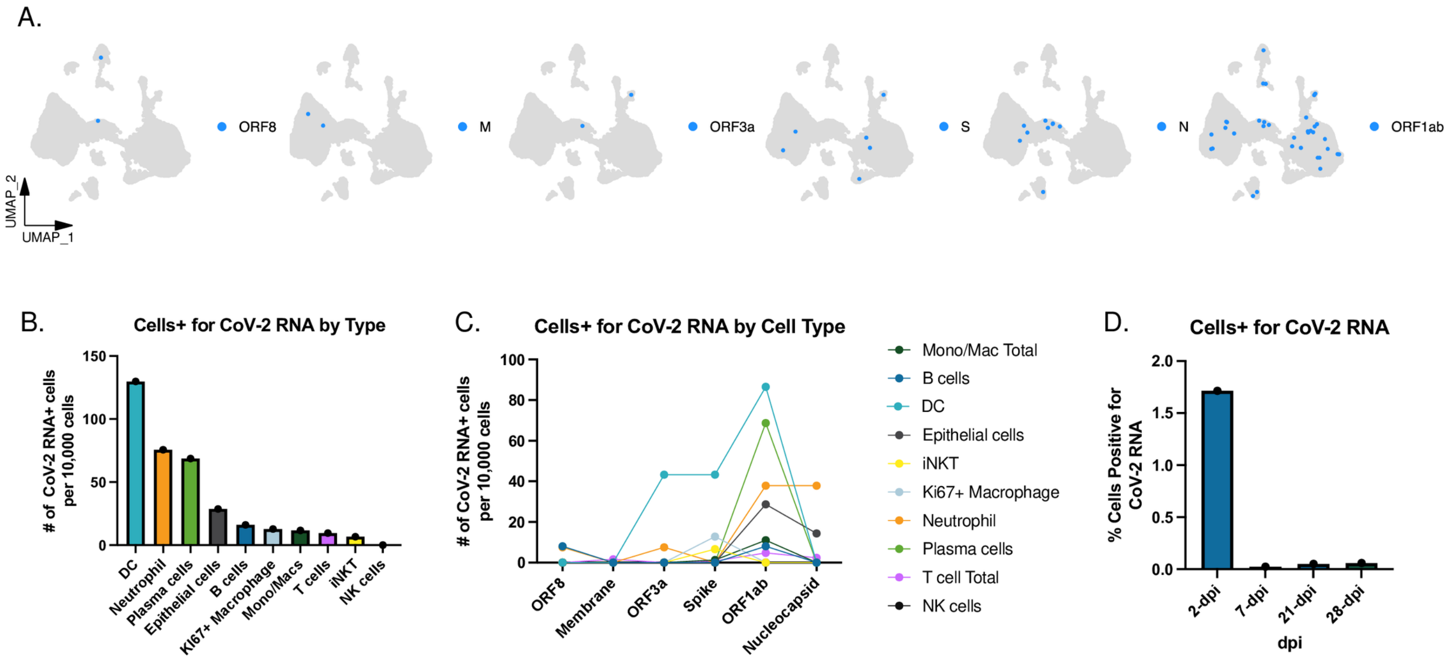

Supplemental Figure 6. Single-cell analysis of SARS-CoV-2 positive cells in BAL of SIV+ PTM. A. UMAP plots highlighting cells with detectable SARS-CoV-2 transcripts. **B.** Distribution of cells by cell type positive for any SARS-CoV-2 transcript. **C.** Number of cells positive for specific SARS-CoV-2 transcripts grouped by cell type. **D.** Percentage of cells positive for SARS-CoV-2 transcripts by days post-infection (dpi).

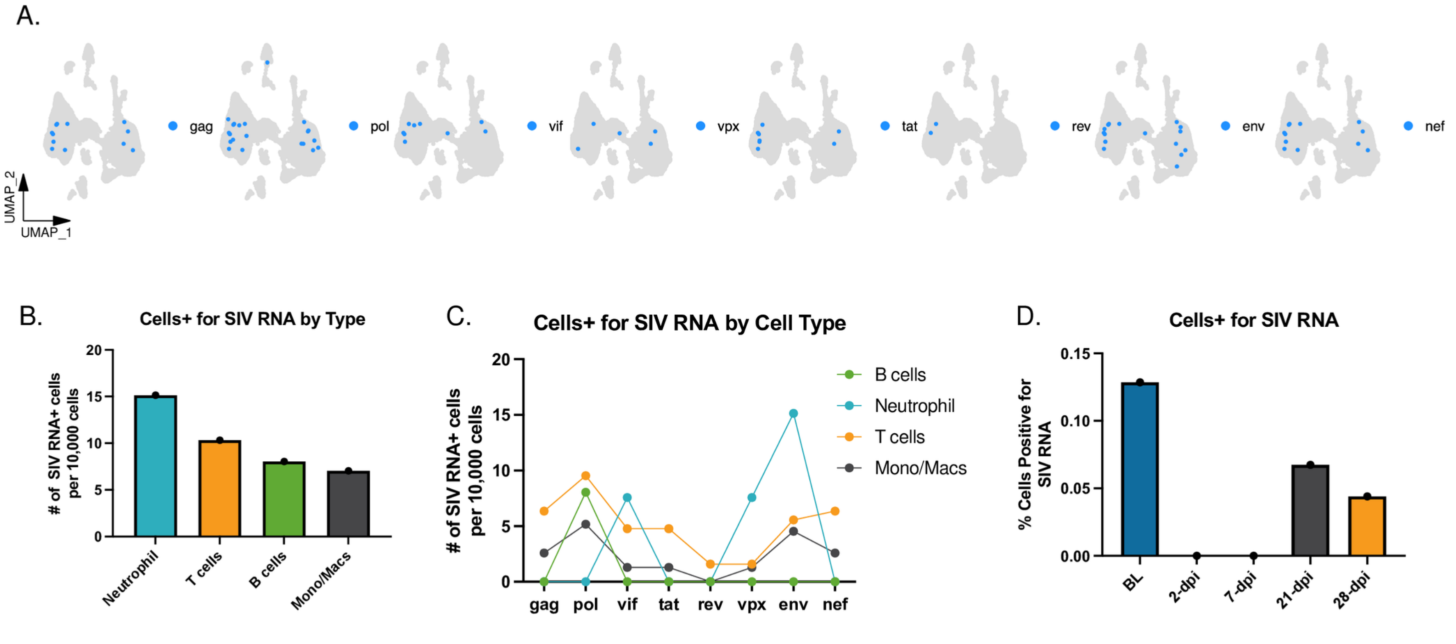

Supplemental Figure 7. Single-cell analysis of SIV positive cells in BAL of coinfected PTM. A. UMAP plots highlighting cells with detectable SIV transcripts. **B.** Distribution of cells grouped by cell exhibiting specific SIV transcripts. **C.** Number of cells positive for specific SIV transcripts grouped by cell type. **D**. Percentage of cells positive for SIV transcripts by days exposure.

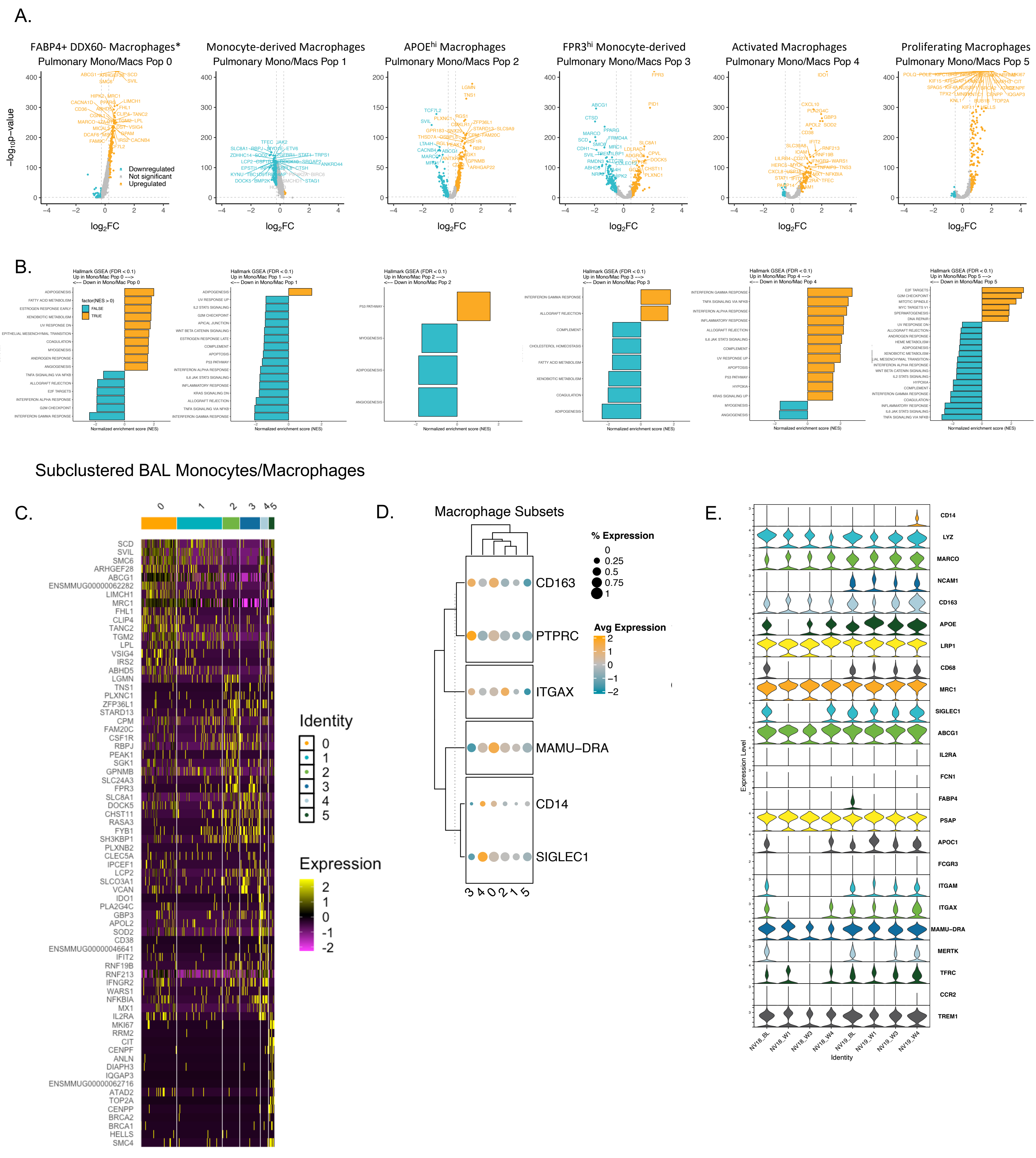

A.
FABP4+ DDX60- Macrophages*
Pulmonary Mono/Macs Pop 0
Monocyte-derived Macrophages
Pulmonary Mono/Macs Pop 1
APOEhi Macrophages
Pulmonary Mono/Macs Pop 2
FPR3hi Monocyte-derived
Pulmonary Mono/Macs Pop 3
Activated Macrophages
Pulmonary Mono/Macs Pop 4
Proliferating Macrophages
Pulmonary Mono/Macs Pop 5
−log10P−value
log2FC
Downregulated
Not significant
Upregulated
B.
Hallmark GSEA (FDR < 0.1)
Up in Mono/Mac Pop 0 --->
<--- Down in Mono/Mac Pop 0
factor(NES > 0)
FALSE
TRUE
Gene set
Normalized enrichment score (NES)
Subclustered BAL Monocytes/Macrophages
C.
Identity
Expression
SCD
SVIL
SMC6
ARHGEF28
ABCG1
ENSMMUG00000062282
LIMCH1
MRC1
FHL1
CLIP4
TANC2
TGM2
LPL
VSIG4
IRS2
ABHD5
LGMN
TNS1
PLXNC1
ZFP36L1
STARD13
CPM
FAM20C
CSF1R
RBPJ
PEAK1
SGK1
GPNMB
SLC24A3
FPR3
SLC8A1
DOCK5
CHST11
RASA3
FYB1
SH3KBP1
PLXNB2
CLEC5A
IPCEF1
LCP2
SLCO3A1
VCAN
IDO1
PLA2G4C
GBP3
APOL2
SOD2
CD38
ENSMMUG00000046641
IFIT2
RNF19B
RNF213
IFNGR2
WARS1
NFKBIA
MX1
IL2RA
MKI67
RRM2
CIT
CENPF
ANLN
DIAPH3
IQGAP3
ENSMMUG00000062716
ATAD2
TOP2A
CENPP
BRCA2
BRCA1
HELLS
SMC4
D.
Macrophage Subsets
% Expression
Avg Expression
CD163
PTPRC
ITGAX
MAMU−DRA
CD14
SIGLEC1
E.
CD14
LYZ
MARCO
NCAM1
CD163
APOE
LRP1
CD68
MRC1
SIGLEC1
ABCG1
IL2RA
FCN1
FABP4
PSAP
APOC1
FCGR3
ITGAM
ITGAX
MAMU−DRA
MERTK
TFRC
CCR2
TREM1
Expression Level
Identity
NV18_BL
NV18_W1
NV18_W3
NV18_W4
NV19_BL
NV19_W1
NV19_W3
NV19_W4

Supplementary Figure 8. Single-cell monocyte/macrophage characterization. A. Volcano plots displaying significantly upregulated and downregulated differentially expressed genes (DEGs) in the Monocyte/macrophage populations. **B.** Bar plots depicting normalized net enrichment score (NES) from gene set enrichment analysis (GSEA) of Hallmark biological pathways. A false discovery rate (FDR) cutoff of 0.1 was used to determine significance. **C.** Heatmap of top 10 differentially expressed genes for each monocyte/macrophage cluster. **D.** Dot plot illustrating gene expression differences among the Seurat-derived clusters. Hierarchical clustering (hclust) was used for column and row clustering. Dot color represents relative gene expression (Rel Expression), while dot size indicates the percentage of cells expressing the gene (% Expression). **E.** Stacked violin plot illustrating gene expression patterns of monocytes/macrophages at baseline (BL) and weeks 1, 3, and 4 post-SARS-CoV-2 infection.

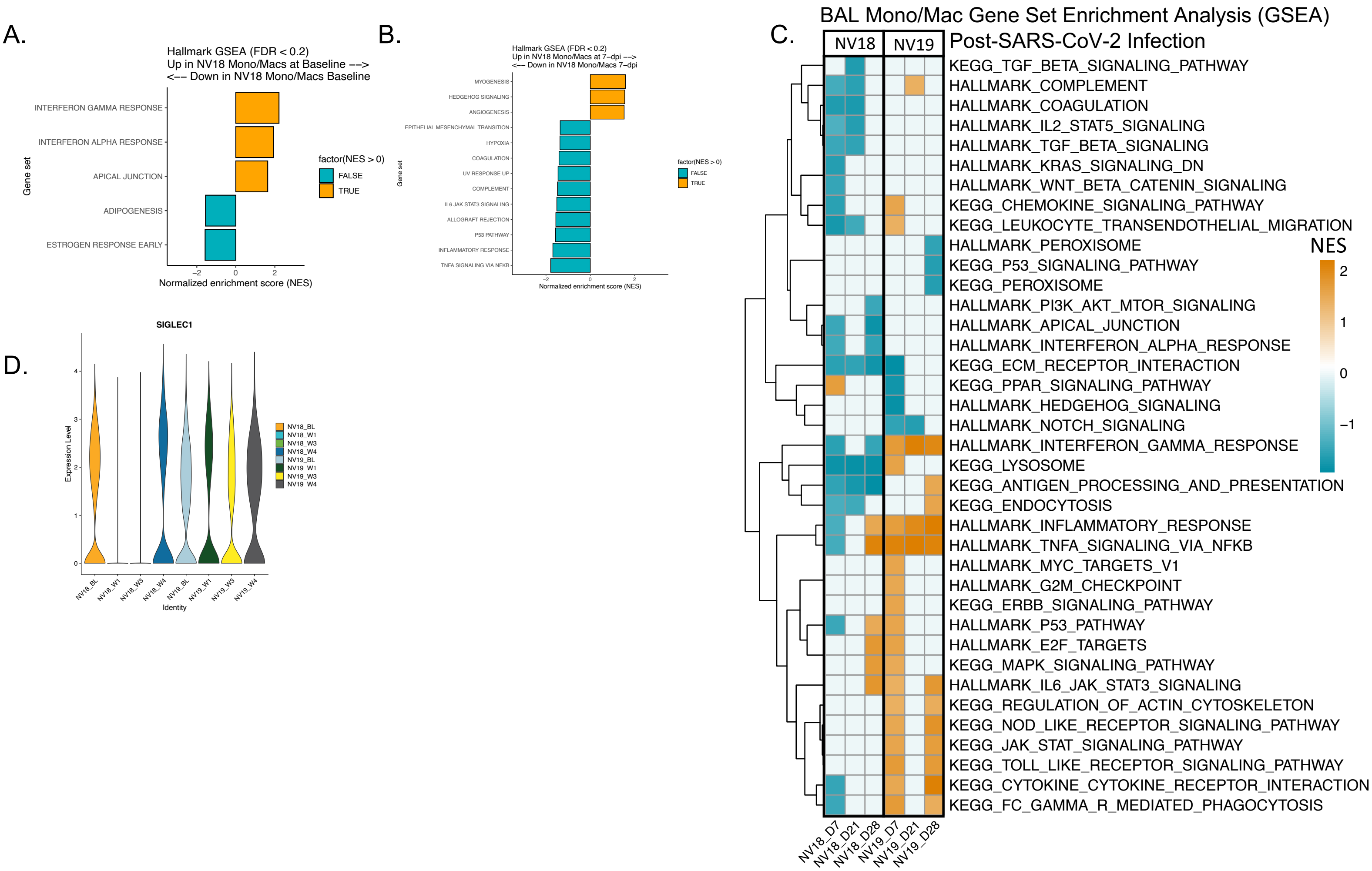

Supplemental Figure 9. Differential gene expression (DEG) and enrichment analysis of Monocyte/Macrophage Subsets in NV18 and NV19. A&B. Gene set enrichment analysis (GSEA) of DEGs comparing NV18 and NV19 at baseline (**A**) and 7-dpi (**B**). **C.** GSEA results comparing DEGs at days 7, 21, and 28 post-exposure to baseline. A false discovery rate (FDR) cutoff of 0.2 was used to determine significance. **D.** CD169 (*SIGLEC1*) expression kinetics.
